# A Mathematical Model on the Resolution of Extrusion Bioprinting for the Development of New Bioinks

**DOI:** 10.3390/ma9090756

**Published:** 2016-09-06

**Authors:** Ratima Suntornnond, Edgar Yong Sheng Tan, Jia An, Chee Kai Chua

**Affiliations:** Singapore Centre for 3D Printing, School of Mechanical and Aerospace Engineering, Nanyang Technological University, Singapore 639798; TA0002AR@e.ntu.edu.sg (E.Y.S.T.); AnJia@ntu.edu.sg (J.A.); MCKCHUA@ntu.edu.sg (C.K.C.)

**Keywords:** extrusion system, bioprinting, 3D printing, pluronic F-127, bioink

## Abstract

Pneumatic extrusion-based bioprinting is a recent and interesting technology that is very useful for biomedical applications. However, many process parameters in the bioprinter need to be fully understood in order to print at an adequate resolution. In this paper, a simple yet accurate mathematical model to predict the printed width of a continuous hydrogel line is proposed, in which the resolution is expressed as a function of nozzle size, pressure, and printing speed. A thermo-responsive hydrogel, pluronic F127, is used to validate the model predictions. This model could provide a platform for future correlation studies on pneumatic extrusion-based bioprinting as well as for developing new bioink formulations.

## 1. Introduction

Bioprinting is an emerging technology that integrates robotic dispenser with multi-materials and living materials. It has brought many benefits to tissue engineering and other biomedical applications by fabricating customized tissue constructs and prostheses [1,2,3]. A typical bioprinting system consists of three components including: (i) living materials which mostly refer to cells; (ii) hydrogel or bioink; and (iii) a bioprinter [4,5]. For bioprinters, there are mainly three types: laser-based bioprinters, ink-jet-based bioprinters, and extrusion-based bioprinters [6,7]. Among the three bioprinting systems, an extrusion-based bioprinter has the highest capability to deal with the widest range of biomaterials at a viscosity range from 30 to 6 × 10^8^ mPa.s [8,9,10]. Hence, an extrusion-based bioprinter is considered one of the most versatile techniques and has been extensively used for biomedical applications [11,12,13,14,15]. Furthermore, recent research demonstrates that electrospinning process can also be re-designed to have extrusion-like capability for the controlled printing of polymers and cells [16,17].

Bioink or hydrogel serves as a support material for cells and is one of the most important components in an extrusion bioprinting system. Many synthetic and natural hydrogels have been used in bioprinting (as bioink) and biofabrication, such as polyethylene glycol (PEG) block polymer, hyaluronic acid, collagen, and alginate [4,18,19]. However, few research works have been trying to understand and predict how the properties of bio-ink and the printing parameters relate to the final form of the printed construct. For example, Bruneaux et al. (2008) showed how viscosity relates to shear rate inside the nozzle and how applied pressure relates to materials flow rate [20]. This work provides a basic understanding of a micro-extrusion bioprinting system. Cheng et al. (2008) focused on cell-laden hydrogel behavior and cell viability on extrusion [21]. This interesting work showed the relationship between cell viability and shear stress, but not the relationship between viscosity and resolution of the printed scaffold. Lee et al. (2015) developed a mathematical model on the relationship between printing speed and resolution; nonetheless, important parameters such as applied pressure and materials viscosity are not considered in the model [22].

Therefore, there is a need of further development on how materials properties and printing parameters affect the printed width (resolution) of hydrogel construct, as it reflects the shape fidelity and complexity that the system can achieve. In this work, we have selected pluronic F127 as model material and developed a simple yet accurate model to understand the influence of printing parameters on a hydrogel strand. In particular, how the printing resolution is related to the size of the nozzle, stage moving speed, and gauge pressure to print a continuous hydrogel strand is examined.

## 2. Materials and Methods 

### 2.1. Pluronic F127 Hydrogel Preparation

Pluronic F127 (P2443) was purchased from Sigma-Aldrich, USA. Pluronic F127 is in white powder form. In order to make 24.5 wt % pluronic hydrogel, 12.25 g of pluronic were mixed with 50 mL of deionized (DI) water under vigorous stirring at 4 °C. For 30 wt % pluronic hydrogel, 15 g of pluronic were mixed with 50 mL DI water under vigorous stirring at 4 °C. After the solution was mixed and became homogeneous, 5 mL of pluronic hydrogel were poured into a syringe before the actual printing.

### 2.2. Rheological Characterization

In order to develop the pressure model, a rheological study was conducted at first. A 40-mm, 2.022° cone plate rheometer (DHR, TA Instruments) was used to obtain the power law index (n) of the prepared pluronic F127 hydrogel. From the work of Abdel-Hamid et al. (2006), the power law model was the best fit for pluronic compared to the Bingham and Casson model [23]. Thus, in this work, we chose the power law model for the rheological study which showed a good fit at R^2^ = 0.9962 (please see details in Supplementary Materials). When the reference shear rate ($\gamma_{0}$) = 1 s^−1^, the relationship of viscosity, shear rate, and power law index can be described in Equation (1) below [22]:

(1) $$Ƞ=\;{Ƞ}_{0}({\dot{\gamma )}}^{n-1},$$

where η is apparent viscosity, η_0_ is zero viscosity, and $\dot{\gamma }$ is shear rate. In order to find the power law index (n), both sides of Equation (1) were changed into log $\dot{\gamma }$ form to obtain a linear region.

(2) $$\text{log}\;Ƞ=\left(n-1\right)\text{log}\;\dot{\gamma }+\text{log}\;{Ƞ}_{0}.$$

**Figure 1:**
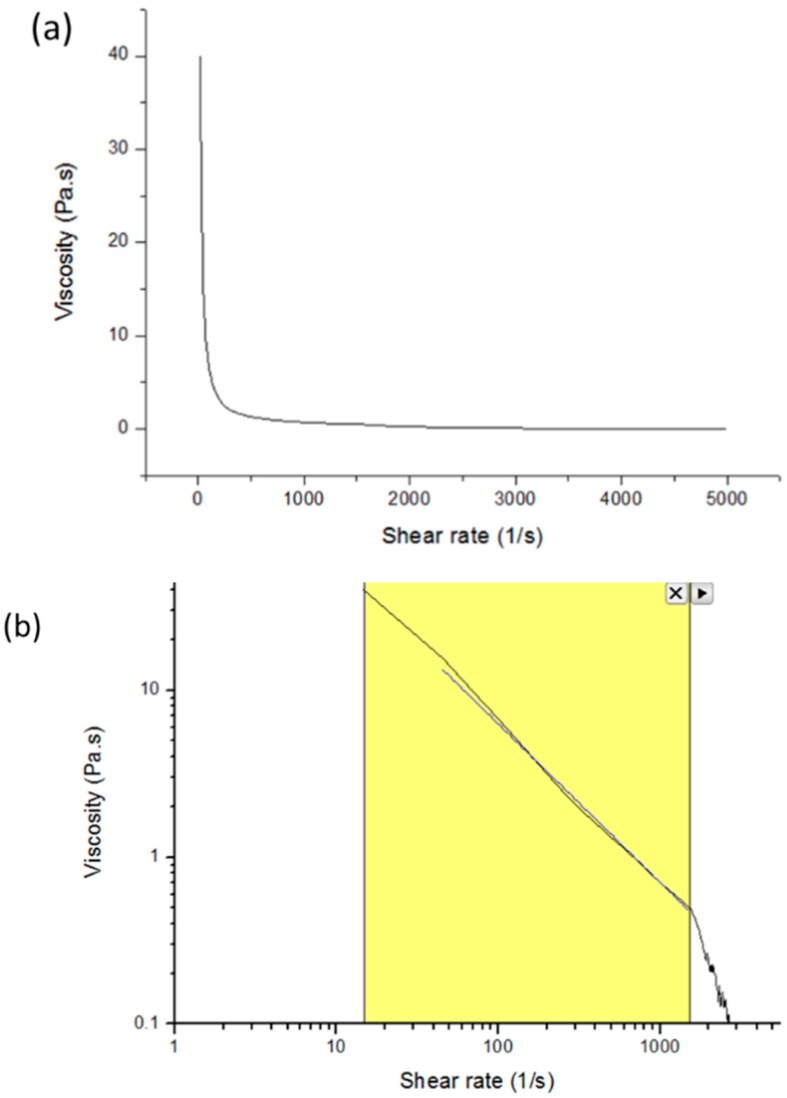
(**a**) Viscosity vs. shear rate cure and (**b**) viscosity and shear rate in linear region for curve fitting (yellow region) in log-log scale.

From rheometer, we obtained the relationship of viscosity and shear rate for 24.5 wt % pluronic hydrogel. As shown in Figure 1a, pluronic viscosity decreases when shear rate increases, which shows that pluronic is a shear thinning polymer, and the power law index (n) can be found from curve fitting in Figure 1b at the shear rate that was in a linear region from start to 1500 s^−1^ shear rate, in which n = 0.0511 (from Figure S1).

### 2.3. Resolution Study Based on Three Different Factors

#### 2.3.1. Pluronic F127 Hydrogel Printing

For 24.5 wt % pluronic, three different nozzle sizes were used, 21G (inner diameter: 514 μm), 25G (inner diameter: 260 μm), and 27G (inner diameter: 210 μm). Three different levels of pressure (1, 2, and 3 × 10^5^ Pa) and three levels of stage moving speed (0.01, 0.02, and 0.03 m/s) were studied in this printing experiment by using pneumatic extrusion-based bioprinter (Regenhu, Villaz-St-Pierre, Switzerland). A total of 27 (3 × 3 × 3) experiments were investigated. Hydrogel was printed onto a clear glass slide, which is the platform for holding the structure. For 30% pluronic, two different nozzle sizes were used: 21G and 25G. The stage moving speed at 0.03 m/s and six different pressure conditions were studied for model verification.

#### 2.3.2. Pluronic F127 Hydrogel Characterization

The widths of the printed lines in all 27 experiments were measured by using an optical microscope (Carl Zeiss, Oberkochen, Germany) at 5× magnification. In each experiment, three lines were printed continuously.

### 2.4. Mathematic Modeling of Line Width, Gauge Pressure, and Stage Moving Speed

**Figure 2:**
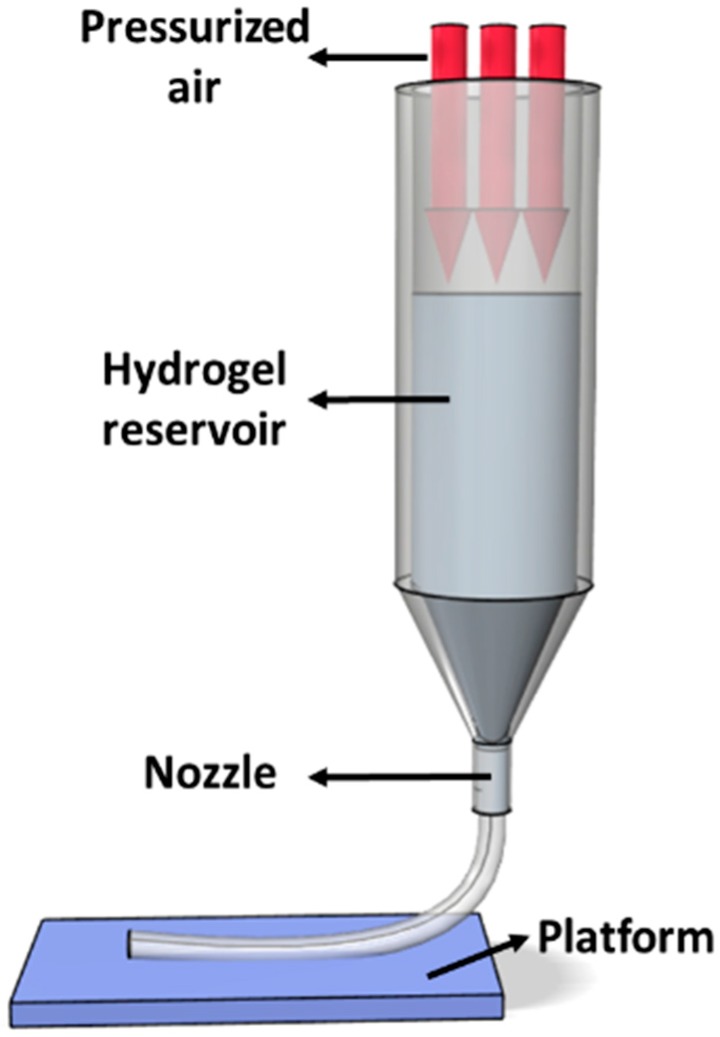
Schematic of pneumatic extrusion.

As shown in Figure 2, the pneumatic extrusion dispenser system is similar to capillary rheometer [20,21,24], which can be modeled by the following equations.

First, the maximum stress in capillary flow ($\tau_{w}$) can be found from

(3) $$\tau_{w\;}=\;\frac{R∆P}{2L}\;=\;\frac{D∆P}{4L},$$

where R is the radius of the nozzle, D is the diameter or size of the nozzle, L is the length of the nozzle, and ∆P is the pressure drop through the reservoir and nozzle.

The shear rate in capillary of power law flow (${\dot{\gamma }}_{\text{w}}$) can be found from

(4) $${\dot{\gamma }}_{\text{w}}\;=\;\frac{3n\;+\;1}{4n}\cdot \frac{4\dot{Q}}{{\pi R}^{3}}\;=\;\frac{3n\;+\;1}{4n}\cdot \frac{32\dot{Q}}{{\pi D}^{3}},$$

where $\dot{Q}$ is the volumetric flow rate.

Assuming the geometry of the printed hydrogel strand is constant from the start point to the end point and the strand is printed out in continuous manner, the flow rate $\dot{Q}$ is equal to

(5) $$\dot{Q}=\frac{{\pi d}^{2}}{4}\;\dot{v},$$

where d is the printed strand diameter and $\dot{v}$ is the stage moving speed.

If we substitute $\dot{Q}$ in Equation (5) into Equation (4), we obtain

(6) $${\dot{\gamma }}_{\text{w}}\;=\;\frac{3n+1}{4n}\cdot \frac{8d^{2}\dot{v}}{D^{3}}.$$

From apparent viscosity, we can find the relationship between stress and shear rate, which is

(7) $$\tau_{w}=\;Ƞ{\dot{\gamma }}_{\text{w}}.$$

If we substitute $\tau_{w}$ from Equation (3) and ${\dot{\gamma }}_{\text{w}}$ from Equation (6) into Equation (7), we obtain

(8) $$P\;=\;32ȠL\left(\frac{3n\;+\;1}{4n}\right)\cdot \frac{d^{2}}{D^{4}}\cdot \dot{v}.$$

Therefore,

(9) $$d^{2}=\frac{D^{4}}{32ȠL\dot{v}}\cdot \left(\frac{4n}{3n+1}\right)\cdot ∆P,$$

or

(10) $$d\left(\dot{v},∆P\right)=D^{2}.\sqrt{\frac{1}{32ȠL\dot{v}}\cdot \left(\frac{4n}{3n\;+\;1}\right)\cdot ∆P},$$

where ∆P is the gauge pressure (Pa).

Thus, the relation between printed hydrogel width (resolution, d), pressure (∆P), stage moving speed ($\dot{v}$), and nozzle size (D) can be described as shown below.

At a constant nozzle size,

$$d\;\propto \;\sqrt{\frac{1}{\dot{v}}}\;\text{and}\;d\propto \sqrt{∆P}\;\text{or}$$

At a different nozzle size.

$$d\;\propto {\;D}^{2}\sqrt{\frac{1}{\dot{v}}}\;\text{and}\;d\propto D^{2}\sqrt{∆P}$$

Table 1 shows the constants that are used for verifying Equation (10) (ΔP and D values are presented in the supplementary file (Table S1 and S2). The power law index and apparent viscosity were determined from rheological study. The needle length was measured from the needle purchased from Nordson EFD (East Providence, RI, USA), and the atmospheric pressure is at standard pressure. 

## 3. Results and Discussion

### 3.1. Resolution Study on a Pneumatic Extrusion-Based Bioprinter 

**Figure 3:**
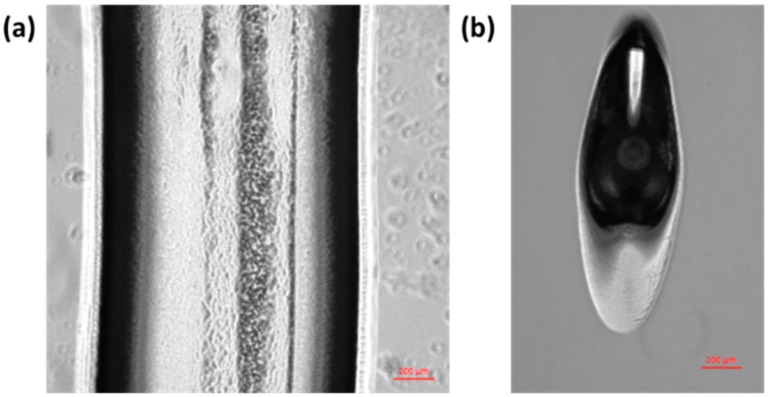

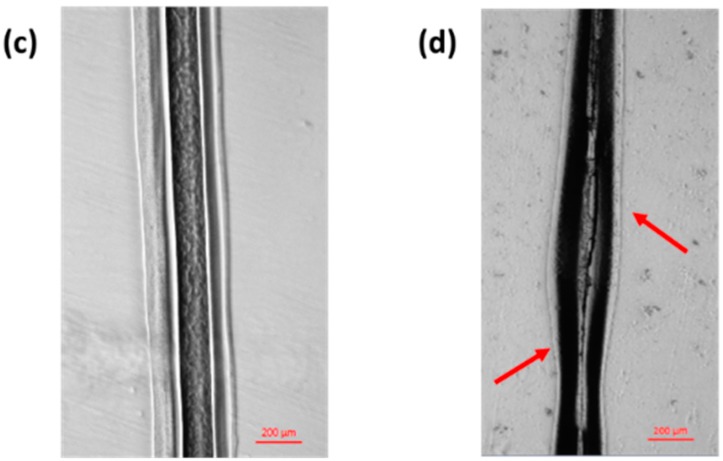
Pluronic hydrogel printed strand under 5× magnification optical microscope at (**a**) 25G nozzle, 3 × 10^5^ Pa, 0.01 m/s speed; (**b**) 27G nozzle, 3 × 10^5^ Pa, 0.01 m/s speed; (**c**) 21G nozzle, 1 × 10^5^ Pa, 0.02 m/s speed; (**d**) 21G nozzle, 1 × 10^5^ Pa, 0.03 m/s speed, where red arrows point to the line defects (scale bar = 200 μm).

Table 2 shows the line width of printed pluronic hydrogel strands at various nozzle sizes, printing speeds, and gauge pressures. From the results, it can be seen that, in order to get a continuous line, the pressure must be sufficient to dispense a continuous strand at the given speed. However, when there is too much pressure (at operation pressure 3 × 10^5^ Pa of 21G nozzle size), it is obvious that the width of hydrogel strand becomes larger than the nozzle size (514 μm). Similarly, the results of 25G show that hydrogels can be printed continuously at 3 × 10^5^ Pa, but the pressure is too high, leading to a width larger than the nozzle size (260 μm), as shown in Figure 3a. Surprisingly, the line width result from 27G nozzle at 3 × 10^5^ Pa with a 0.01-m/s speed is also wider than the nozzle size. This is possibly due to the fact that pluronic was printed in discrete elongated droplets instead of a continuous strand, as shown in Figure 3b. The discontinuous strand may result from the surface tension that becomes dominant after extrusion. However, in this experiment, pluronic F127 at 24.5% w/v was used, which is a high-viscosity hydrogel (1.04 Pa.s or 1040 cP). Hydrogel that has a high viscosity may be able to overcome the effect of surface tension [25]. Therefore, for the mathematical modeling, this phenomenon is not included in the model. These elongated droplets are typically wider than a straight line.

Another interesting observation is that, at a higher stage moving speed, the printed hydrogel has more defects compared to the hydrogel line that was printed at a lower speed, as shown in Figure 3c,d. By using a 21G nozzle at the highest stage moving speed, the thinnest line can be fabricated. This is similar to the finding from the work of Müller et al. who used a micro-valve-based nozzle to print pluronic [11]. Even though a finer line had been obtained, it was not suitable to print a complex structure at this speed (0.03 m/s). The defects at the first layer will not allow the build-up of a 3D structure. Rather, a moving speed of 0.02 m/s is more suitable to print a continuous uniform hydrogel strand (Figure 3c).

### 3.2. Model Verification

**Figure 4:**
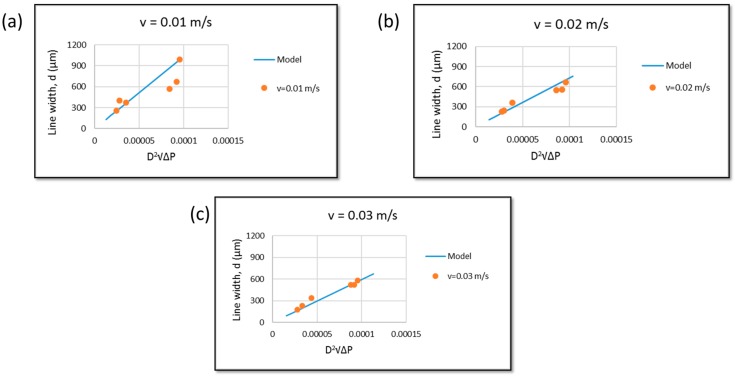
Relationship of d vs. $D^{2}\sqrt{∆P}$ of experimental data and theoretical data at different condition: (**a**) $\dot{v}$ = 0.01 m/s; (**b**) $\dot{v}$ = 0.02 m/s and (**c**) $\dot{v}$ = 0.03 m/s.

Dispensing pluronic F127 hydrogel with different nozzle sizes on the pneumatic extrusion bioprinter at different stage moving speeds ($\dot{v}$) produces different results. As shown in Figure 4, both theoretical and experimental line widths that represent resolution were plotted. It shows the relationship between pressure and hydrogel strand width of the model and the theoretical data at different stage moving speeds. The aforementioned model was developed with the following assumptions: (i) no slip at wall; (ii) fluid is incompressible; (iii) flow is steady and laminar; (iv) no change in strand cross sectional surface; and (v) the pressure drop at entrance and exit are negligible.

This model does not include the effect of temperature and degree of printed strand continuality. For a speed of $\dot{v}$ = 0.01 m/s, some experimental data are close to the theoretical data, but the off data points made the overall trend different from the model (Figure 4a). On the other hand, as shown in Figure 4b,c, the experimental data from $\dot{v}$ = 0.02 m/s and $\dot{v}$ = 0.03 m/s have some values that stay in the region between the predicted models. Nevertheless, they still show similar trends compared to the theoretical data. Figure 4 shows the experiment data following the model trend, which is $\;d\propto D^{2}\sqrt{∆P}$.

**Figure 5:**
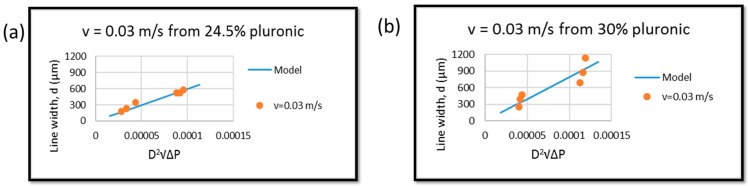
Relationship of d vs. $D^{2}\sqrt{∆P}$ of experimental data and theoretical data at different concentrations at $\dot{v}$ = 0.03 m/s: (**a**) 24 wt % pluronic and (**b**) 30 wt % pluronic.

Even though pluronic can gel at 20 wt %, due to the micelle packing network, pluronic is commonly printed at 24.5 wt % or higher [26,27]. Thus, two concentrations of pluronic (24.5 wt % and 30 wt %) were used and compared. As shown in Figure 5, this model proved that it can work with different concentrations. The data points followed the model trend, though some errors occurred. In a future work, the correction factor should be included to minimize this error.

Though the theoretical results show similar trends, they still show some errors. This error may be due to the change in the effect of pressure during entrance to, and exit from, the nozzle. Moreover, there is a change in pressure from the syringe barrel to the nozzle, which may affect the results as well. As shown in Figure 4, the error is reduced at a higher speed, which may be due to the fact that this bioprinter is a pneumatic system and it always has a pressure drop [25], which results in material returning to its original viscosity, owing to the thixotropic properties of materials [28]. However, at a higher speed, less time is needed to allow material to recover. Therefore, viscosity is constant at higher speeds. Moreover, the die swell of hydrogel might be another reason for the error between actual results and the model [29]. However, Feilden et al. (2016) showed that pluronic has shear thinning properties, which eliminates the die swell problem and allows the filament to emerge smoothly [30]. Therefore, in this work, the die swell is not included in the model. Lastly, the change in geometry of the hydrogel strand upon its exit from the nozzle may also contribute to the error, as one study reported that the deviation could be due to the internal diameter of the nozzle (needle) lack of uniformity [31]. A correction factor may be added into the model to eliminate this error.

**Figure 6:**
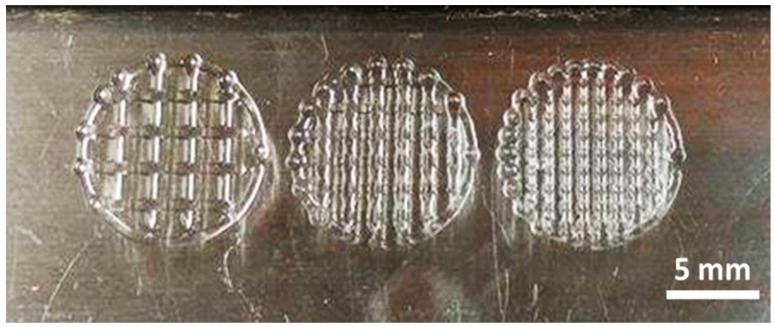
Grid structure (single layer) of pluronic F127 by using 21G nozzle at 1 × 10^5^ Pa and 0.025 m/s speed (scale bar = 5 mm).

The objective of this research is to develop a simple yet accurate mathematical model to predict the line width of hydrogel strand at a given speed, the minimum gauge pressure required, and the nozzle size. Moreover, by understanding how each parameter influences the width of dispensed hydrogel in a pneumatic-based bioprinter system, an optimum condition can be found to obtain a complex hydrogel structure with good resolution, as shown in Figure 6 (at optimum condition, grid pattern at different spacing from 2 mm to 0.5 mm (from left to right) can be fabricated). However, this model is unable to predict and incorporate the sol-gel transition behavior of thermos-responsive hydrogels, which have properties that affect viscosity of the hydrogels. In order to investigate the hydrogel properties effect on printability, nozzle size should be kept constant to deeply explore significant effects. In fact, pluronic F127 has a good printability at a range of viscosities [11,27]. Although synthetic polymers are relatively easy to print and can be biocompatible [32], they are usually not bioactive and may not be suitable for long-term cell culture [18]. Recently, Kolesky et al. showed that, by extruding pluronic as a sacrificial mold, gelatin methacrylate (GelMA) constructs with micro-channels can be fabricated, which could be potentially useful for vascularized tissue engineering [13]. Nevertheless, the resolution achievable from pneumatic extrusion-based bioprinter still remains elusive [33]. In this research, we modeled the relationship of extrusion-based bioprinting parameters by using thermo-responsive hydrogel pluronic F127. Pluronic F127 is the hydrogel that the resolution can be determined directly after printing. Other hydrogels, for example, alginate which is crosslinked by a ionic crosslink reaction. The final resolution still depends on the swelling after the alginate reacts with a divalent ion such as Ca^2+^ and Ba^2+^ [34]. On the other hand, GelMA is thermo-responsive and a UV-crosslinked hydrogel, and the final resolution of GelMA can only be determined after a UV curing process. This process may lead to shrinkage of the GelMA hydrogel strand [35]. In this work, the gelation mechanism and the effect of post-processing on the hydrogel strand were not included in the model. Hence, this analytical model needs to be further developed in order to be applied to other types of thermo-responsive hydrogels or even cell-laden hydrogel, which have been used extensively for tissue engineering and other biomedical applications, for predicting optimal processing conditions. 

## 4. Conclusions

A simple yet accurate analytical model has been developed for predicting the resolution of extrusion bioprinting. Pluronic F-127 at 24.5 wt % was printed by using a pneumatic extrusion-based bioprinter, and the width of printed hydrogel strand was measured against the size of the nozzle, pressure, and stage moving speed. Experimental data showed a good agreement with model predications, despite some errors possibly due to the capillary entrance and hydrogel surface tension effects. The knowledge obtained from this research may help develop new pluronic-based bioink formation and optimize bioprinting parameters. 

## Figures and Tables

**Table 1:** Constant values for mathematical model verification.

Pluronic Concentration	24.5 wt %	30 wt %
Power law index (n)	0.0511	0.1656
Apparent viscosity (Ƞ)	1.04 * Pa.s	1.46 Pa.s
Needle length (L)	0.005 m	0.005 m

* See details in Supplementary Materials.

**Table 2:** Line width results from extrusion-based bioprinter in micrometers (μm).

**Nozzle Size**	**Stage Moving Speed (m/s)**	**Gauge Pressure × 10^5^ (Pa)**		
		**1**	**2**	**3**
**21G (514 μm)**	**0.01**	566.17 ± 14	2668.05 ± 10	3215.85 ± 24
	**0.02**	368.75 ± 7 ^b^	1771.66 ± 30	2237.90 ± 20
	**0.03**	325.48 ±2 ^b^	1512.28 ± 7	1852.55 ± 15
**25G (260 μm)**	**Stage Moving Speed (m/s)**	**Pressure × 10^5^ (Pa)**		
		**1**	**2**	**3**
	**0.01**	N/A ^a^	325.06 ± 17 ^b^	1447.95 ± 1
	**0.02**	N/A ^a^	286.72 ± 33 ^b^	527.68 ± 19 ^b^
	**0.03**	N/A ^a^	220.72 ± 23 ^b^	406.20 ± 13 ^b^
**27G (210 μm)**	**Stage Moving Speed (m/s)**	**Pressure × 10^5^ (Pa)**		
		**1**	**2**	**3**
	**0.01**	N/A ^a^	254.69 ± 9 ^b^	435.47 ± 7 ^b^
	**0.02**	N/A ^a^	N/A ^a^	385.46 ± 11 ^b^
	**0.03**	N/A ^a^	N/A ^a^	281.86 ± 18 ^b^

^a^ No hydrogel was printed out (not sufficient applied pressure); ^b^ Not continuous strand (defect strand).

## Supplementary Materials

The following are available online at www.mdpi.com/1996-1944/9/9/756/s1.
