# Supplementary material for: A Mathematical Model on the Resolution of Extrusion Bioprinting for the Development of New Bioinks
Source: Materials (Basel). 2016 Sep 6;9(9):756. doi: 10.3390/ma9090756 (PMC5457067; doi:10.3390/ma9090756)
Supplement: Supplementary file 1 [file materials-09-00756-s001.pdf]

Supplementary Materials: A Mathematical Model on the Resolution of Extrusion Bioprinting for the Development of New Bioinks

Ratima Suntornnond, Edgar Yong Sheng Tan, Jia An and Chee Kai Chua

Table S1. Gauge pressure (ΔP) and Nozzle diameter (D) value used in Equation (10) for 24.5 wt % Pluronic.

Nozzle Gauge	Needle Diameter, D (m)	Gauge Pressure, ΔP × 10^5 (Pa) Stage Moving Speed		
		**0.01 m/s**	**0.02 m/s**	**0.03 m/s**
21G	0.000514	1.26	1.52	1.80
25G	0.000260	2.50	3.00	3.60
27G	0.000210	3.10	3.71	4.46

Table S2. Gauge pressure (ΔP) and Nozzle diameter (D) value used in Equation (10) for 30 wt % Pluronic.

Nozzle Gauge	Needle Diameter, D (m)	Gauge Pressure, ΔP × 10^5 (Pa) Stage Moving Speed		
		**0.01 m/s**	**0.02 m/s**	**0.03 m/s**
21G	0.000514	1.77	2.12	2.50
25G	0.000260	3.50	4.20	5.04
27G	0.000210	4.33	5.20	6.24

Apparent Viscosity (ƞ)

We would like to minimize the number of printing variables by first determining the material's viscosity which is considered constant and has been used for the theoretical model.

The shear rate in capillary of power law flow ($\dot{\gamma}_w$) can be found from

$$\dot{\gamma}_w = \frac{3n+1}{4n} \cdot \frac{4\dot{Q}}{\pi R^3} = \frac{3n+1}{4n} \cdot \frac{32\dot{Q}}{\pi D^3} \quad (S1)$$

where $\dot{Q}$ is volumetric flow rate.

Assuming the geometry of the printed hydrogel strand is constant from the start point to the end point and the strand is printed out in continuous manner, the flow rate $\dot{Q}$ equal to

$$\dot{Q} = \frac{\pi d^2}{4}\dot{v} \quad (S2)$$

where d is a printed strand diameter and $\dot{v}$ is stage moving speed.

Substitute $\dot{Q}$ in Equation (5) into Equation (4),

$$\dot{\gamma}_w = \frac{3n+1}{4n} \cdot \frac{8d^2\dot{v}}{D^3} \quad (S3)$$

Assume that at v = 0.03 m/s, there is no change in hydrogel geometry and $d = \frac{D}{2}$, therefore

$$\dot{\gamma}_w = \frac{3n+1}{4n} \cdot \frac{2\dot{v}}{D} \quad (S4)$$

Nozzle size 21G, D = 0.000514 m and n is power law index (n = 0.0511). Substitute D, n and v into Equation (4) $\dot{\gamma}_w = 658.64\ s^{-1}$ or ~660 s^{-1} .

At shear rate 660 s^{-1}, apparent viscosity (ƞ) is around 1.04 Pa.s as shown in Figure S1 below. The viscosity for 30 wt % pluronic was calculated by using the same method.

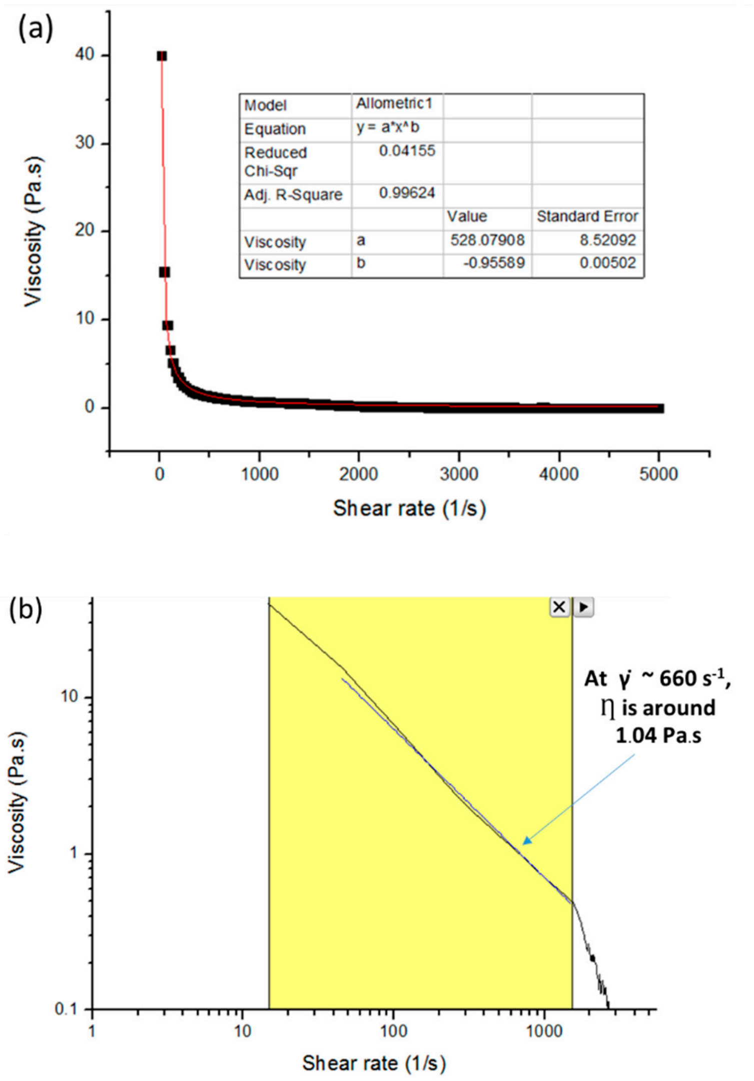

Figure S1. (**a**) Viscosity vs. shear rate cure with curve fitting ($R^2 = 0.99624$) and (**b**) viscosity and shear rate in linear region for curve fitting (yellow region) in log-log scale.
